# Peste des petits ruminants virus (PPRV) modulates caprine dendritic cell function and induces immunosuppression through IL-10 upregulation

**DOI:** 10.1080/21505594.2026.2629119

**Published:** 2026-02-16

**Authors:** Pablo Nogales-Altozano, Abel Martínez-Rodrigo, José M Rojas, Noemí Sevilla

**Affiliations:** a Centro de Investigación en Sanidad Animal, Instituto Nacional de Investigación y Tecnología Agraria y Alimentaria, Consejo Superior de Investigaciones Científicas (CISA-INIA-CSIC), Valdeolmos, Madrid, Spain; b Universidad Autónoma de Madrid, Escuela de Doctorado, Madrid, Spain; c Animal Science Department, Veterinary School, Universidad Complutense de Madrid, Madrid, Spain

**Keywords:** Morbillivirus, IL-10, immunosuppression, dendritic cells, goats

## Abstract

Peste des petits ruminants (PPR) is a WOAH notifiable disease affecting sheep and goats, caused by Peste des petits ruminants virus (PPRV), a morbillivirus of the *Paramyxoviridae* family. Infection with PPRV leads to immunosuppression, creating conditions for opportunistic infections that can result in animal mortality. Although goats generally exhibit more severe clinical signs than sheep, the underlying mechanisms driving this species-specific difference remain poorly understood. Dendritic cells (DC), which play a pivotal role in initiating immune responses, are among the immune targets of PPRV in small ruminants. In this study, we examined the impact of PPRV on caprine immune cells, focusing on CD14^+^ monocytes and monocyte-derived dendritic cells (MoDC). Our findings indicate that PPRV infects goat monocytes without preventing their differentiation into DC. Infected MoDC displayed increased expression of maturation markers and reduced phagocytic activity, suggesting a transition toward an activated phenotype. However, mixed lymphocyte reaction assays revealed that PPRV-infected MoDCs have a diminished capacity to promote T cell proliferation. This impaired function was associated with elevated IL-10 production and reduced conjugation between DCs and T cells. Overall, PPRV infection induces an atypical maturation stage in goat MoDCs, characterized by partial activation but impaired antigen presentation. These findings demonstrate that PPRV-driven modulation of DC function contributes to the immunosuppression observed during PPRV infection in goats.

## Introduction

Morbillivirus infections, including measles, Canine Distemper, and Peste des Petits Ruminants (PPR) viruses, are highly contagious and progress rapidly, causing lesions in the respiratory tract and gastrointestinal tract, as well as in lymphoid organs [1]. Morbillivirus infections often result in acute pneumonia and severe gastroenteritis, leading to immunosuppression characterized by leukopenia and a weakened antibody response [2,3]. These infections usually begin in the respiratory tract, where antigen-presenting cells (APCs) are initially infected by the binding of the hemagglutinin (H) glycoprotein to the CD150 receptor. These infected APCs then migrate to nearby lymph nodes, initiating the first stage of viral replication. The virus subsequently spreads systemically, likely through infected lymphocytes, to other lymphoid tissues [4]. Eventually, Morbilliviruses reach and infect epithelial tissues in the lungs and gastrointestinal tract, where clinical signs such as pneumonia and gastroenteritis manifest and high viral loads are detected [3,5].

APCs play a pivotal role in initiating immune responses by rapidly detecting infected cells and activating downstream immunity. Among APCs, dendritic cells (DCs) are particularly critical due to their unique capacity to initiate primary immune responses. Although they represent only about 1% of circulating mononuclear cells, DCs are highly specialized in capturing, processing and presenting antigens to T cells. Upon encountering viral pathogens, DCs detect pathogen-associated molecular patterns (PAMPs) through their pattern recognition receptors (PRRs), which triggers their activation. This activation enables efficient antigen processing and presentation via major histocompatibility complexes (MHC) type I and II. Importantly, DCs are the only APCs capable of priming naïve T cells [6], making them essential for the induction of adaptive immunity. However, many viruses, including *Morbillivirus* genus, have developed strategies to evade innate immunity recognition to promote their replication [7,8]. Given Morbillivirus immunosuppressive potential and their capacity to infect immune cells, the targeting or dysfunction of DCs during Morbillivirus infection may represent a key strategy for immune evasion and immunosuppression.

A crucial immunosuppressive factor in this context is interleukin-10 (IL-10), a potent anti-inflammatory cytokine frequently upregulated during viral infections [9]. IL-10 plays a pivotal role in modulating immune response by directly targeting DCs, where it inhibits their maturation, downregulates the expression of co-stimulatory molecules and MHC class II, and suppresses the production of pro-inflammatory cytokines. These effects collectively impair the antigen-presenting capacity of DCs and limit their ability to prime naïve T cells, thereby weakening both the initiation and amplification of adaptive immune responses. In the case of *Morbillivirus* infections, the induction of IL-10 may represent a central immune evasion strategy.

Among Morbilliviruses, Peste des Petits Ruminants virus (PPRV) is a highly contagious pathogen that causes a notifiable disease recognized by the World Organization for Animal Health (WOAH). PPRV virions are pleomorphic, have a lipid envelope and vary in size around 500 nm [10]. Their genome is a single-stranded, negative-sense, non-segmented RNA of 15,948 nucleotides [11]. The PPRV genome encodes six structural proteins: nucleoprotein (N), phosphoprotein (P), matrix protein (M), fusion protein (F), hemagglutinin (H) and RNA-dependent RNA polymerase (L), as well as three non-structural proteins: C, V and W [12]. PPRV primarily affects domestic and wild ruminants, with both sheep and goats being the most susceptible hosts [13], but evidence indicates that goats are generally more severely affected than sheep, with higher susceptibility and mortality rates [12,14]. A hallmark of PPRV infection is its immunosuppressive effect, which predisposes animals to secondary opportunistic infections. These co-infections worsen the clinical outcome and significantly increase the economic impact of disease outbreaks. Currently, PPRV is endemic in North, East and West Africa, the Middle East, India, Southeast Asia and China. Notably, between July 2024 and January 2025, the first case of PPR in the European Union was reported in Hungary, Greece and Romania. These outbreaks led to the loss of “PPR-free” status for all three countries, underscoring the urgent need to intensify biosecurity and surveillance measures to prevent the spread of this transboundary disease [15].

A previous study carried out in our laboratory demonstrated that PPRV infection impairs the function of monocytes and DCs in sheep [16], suggesting that these immune cells are key targets of PPRV modulation. However, little is described about how PPRV affects these same immune cells in goats, the species that tend to exhibit more severe disease. Given the central role of DCs in orchestrating immune responses, we hypothesize that differences in the response of monocytes and DCs to PPRV may contribute to the increased susceptibility observed in goats. The aim of this study is to firstly investigate the effects of PPRV infection on caprine monocytes and DCs, and to elucidate the potential mechanisms that could explain the species-specific differences in disease severity. To achieve this, we established a protocol for the generation of caprine monocyte-derived DCs and assessed the impact of PPRV infection on their differentiation and functional capacity in order to elucidate the mechanism that PPRV employs to trigger immunosuppression in DC.

## Materials and methods

### Animals

Five healthy male Murciano-Granadina goats (*Capra Aegagrus*), approximately five years old, were housed at the Department of Animal Reproduction INIA-CSIC animal facility under controlled conditions with free access to food and water. Peripheral blood was collected to isolate peripheral blood mononuclear cells (PBMCs) for subsequent assays.

### Cell lines and viruses

Vero cells expressing the canine SLAM receptor (VDS cells; kindly provided by S. Parida, Pirbright, UK) were maintained in Dulbecco’s Modified Eagle Medium (DMEM; Thermo Scientific) supplemented with 10% fetal bovine serum (FBS; Sigma), L-glutamine, nonessential amino acids, sodium pyruvate, antibiotics, and phleomycin D1 (Zeocin). The virulent PPRV Ivory Coast’89 strain (lineage I), obtained from C.A. Batten (Pirbright, UK), served as the viral source for all experiments. Viral stocks were propagated in VDS cells and titrated using plaque assays as previously described [17,18].

### Caprine monocyte-derived DC isolation and infection

PBMCs were separated by density gradient centrifugation using Ficoll [19]. Briefly, venous blood collected in EDTA (6 mM final concentration) was diluted 1:1 in PBS +0.03% (w/v) EDTA (pH 7.4) and overlayed over a Ficoll cushion (GE Healthcare Europe GmbH, Barcelona, Spain). Blood was centrifuged at 800 × g for 30 min at room temperature without brake, and the PBMC present at the interface were transferred to a fresh tube and washed with PBS +0.03% (w/v) EDTA. Contaminant erythrocytes were lysed and after two further washes PBMCs were obtained for monocyte isolation. For this, a positive selection was performed by magnetic separation using a MACS system (CD14 MicroBeads-human kit, Miltenyi Biotec). Following the protocol provided by the manufacturer, PBMCs were incubated with magnetic spheres bound to anti-CD14 antibody and this was added onto an LS column placed on its magnetized support. In this way, monocytes are retained within the column, and after elution, an enrichment of monocytes higher than 95% was obtained. For antigen presentation experiments, the CD14^−^ fraction was stored in freezing media (90% FBS +10% DMSO) at −80ºC until use. To differentiate monocytes into immature monocyte-derived dendritic cells (iMoDC), cell-enriched fractions were cultured in 12-well plates at 1.5 × 10^6^ cells/well in 1 mL of complete RPMI (RPMIc) (RPMI with 10% FBS, 2% 1 M HEPES, 1% 100x nonessential amino-acids, 1 mM sodium pyruvate, 200 mM L-Gln, 1% penicillin/streptomycin and 0,1% 50 mM 2-mercaptoetanol) supplemented with 20 ng/mL ovine GM-CSF (OvGM-CSF) (Kingfisher Biotech) and 20 ng/mL ovine IL-4 (OvIL-4) (Kingfisher Biotech), and incubated at 37ºC, 5% CO_2_ and > 90% humidity. Cytokines were replenished after 48 h. For infection studies, monocytes or iMoDC were exposed to PPRV at a multiplicity of infection (MOI) of 1 at 0 h or 48 h post-differentiation, respectively. For antigen presentation experiments, iMoDC maturation was induced using the TLR 7/8 agonist R848 (1 µg/ml) (InvivoGen) 16 hours prior to subsequent co-culture.

### Flow cytometry, cell markers, and intracellular staining

Cells were stained with primary and secondary antibodies under standard conditions [16] and fixed with paraformaldehyde. For intracellular staining, cells were fixed and permeabilized with BD Cytofix/Cytoperm™ (BD Biosciences) and then stained with antibody. For intracellular IL-10 (clone CC320; Novus Biologicals) and IFN-γ (clone CC302; Bio-Rad) staining, cells were incubated with Brefeldin-A solution (1 µg/mL; Biolegend) for 3 hours at 37ºC and 5% CO_2_ and stained for surface antigens prior to starting the intracellular staining protocol for cytokines. Antibodies used for characterization of DC are described in [16]. Additionally, we used anti-CD172a (clone DH59B; Bio-Rad) and anti-CD40 (clone IL-A156; Bio-Rad) antibodies for further DC characterization in the present work. For intracellular PPRV detection, anti-PPRV-N monoclonal antibody (gifted by G Libeau, CIRAD, Montpellier, France) was used. Data acquisition was performed on a BD FACSCelestaSORP cytometer and analyzed using the FlowJo v.10 software (Tree Star Inc, USA). Gating strategy for IFN-γ staining is shown in Supplementary Figure S1.

### Apoptosis and phagocytosis assays

Flow cytometry was also used to study apoptosis dynamics, comparing mock- and PPRV-infected iMoDC at 48, and 72 h, with Annexin V and 7-AAD staining using the Annexin V-phycoerythrin (PE) apoptosis detection kit I (BD Biosciences), and following the manufacturer’s instructions. Cells were washed twice with cold PBS and resuspended in 1x Binding Buffer at a concentration of 1 × 10^6^ cells/ml, 100 µl of the cell solution (1 ×10^5^ cells) were transferred to a 5 ml culture tube, followed by the addition of 5 µl of Annexin V-PE and 5 µl 7-AAD. Tubes were gently mixed by vortexing and incubated for 15 min at RT (25°C) in the dark. After incubation, 400 µl of 1x Binding Buffer were added to each tube and analyzed by flow cytometry within 1 hr.

Phagocytosis in monocytes, iMoDC, and mMoDC were quantified by microsphere phagocytosis assays as described in [16]. Flow cytometry analysis was carried out on mock- or PPRV-infected populations to evaluate the percentage of microsphere positive cells and their geometric mean fluorescence intensity (Geo MFI).

### Conjugation assays

MoDC were mock- or PPRV-infected for 48 hours. MoDC were labeled with PKH67 green fluorescent cell linker according to the manufacturer’s instruction (Sigma-Aldrich). Briefly, MoDCs were collected and counted. Cells were washed three times with PBS by centrifugation at 400 × g for 5 min. During the final wash, the PKH67 Green Fluorescent Cell Linker solution was prepared (4 µM PKH67 in Diluent C; equivalent to 1 µL PKH67 in 500 µL Diluent C, sufficient for labeling up to 5 × 10^6^ cells). After aspiration of the supernatant, cells were resuspended in the PKH67 solution and incubated for exactly 5 min at room temperature with gentle mixing to ensure homogeneous labeling. The staining reaction was quenched by adding 2 mL of ice-cold FBS, followed by the addition of 10 mL complete RPMI medium. Cells were pelleted by centrifugation at 400 × g for 5 min, washed twice with 10 mL complete RPMI medium under the same conditions, and finally resuspended in 10 mL complete RPMI medium. Labeled cells were rested for 1 h at 37°C in a humidified atmosphere containing 5% CO_2_ prior to use and pulsed for 10 min with the T cell superantigen Staphylococcal Enterotoxin B (SEB) (1 µg/mL) (Sigma-Aldrich) to facilitate conjugation. The autologous CD14^−^ PBMC fraction was labeled with PKH26 red fluorescent cell linker according to the manufacturer’s instruction (Sigma-Aldrich) as previously described for PKH67. MoDC and CD14^−^ PBMC were co-cultured at 37ºC and fixed with 4% PFA at 0, 3, 5, 10 and 20 minutes prior to analysis by flow cytometry of PKH67^+^ PKH26^+^ doublets indicative of cell conjugation [20].

### Allogenic MLR and proliferation assays

CD14-depleted T cell-enriched fractions were prepared as previously described [17]. Separation efficiency (typically > 70%) was confirmed by flow cytometry. The T cell fraction was then labeled with CellTrace Violet (Thermo Fisher) as described in the manufacturer’s protocol, briefly, T cells were washed 1–2 times in PBS by centrifugation at 400 × g for 6 min at room temperature. Cells were then labeled with CellTrace Violet diluted in PBS at a ratio of 1 µL CellTrace Violet per 2 × 10^6^ cells/mL. The cell suspension was incubated for 15 min at 37°C in a humidified incubator. The staining reaction was quenched by adding five volumes of complete RPMI medium, followed by one wash with PBS under the same centrifugation conditions. The resulting pellet was resuspended in 2 mL complete RPMI medium, and cells were allowed to rest in the dark for at least 15 min at room temperature prior to use, after which they were maintained on ice. Cells were counted immediately after the resting step. After that the cells were cocultured with irradiated (1500 rad) allogenic mock- or PPRV-infected MoDC at different DC/T cell ratios (1:16, 1:8, 1:4 and 1:2) in U-bottom 96-well plates. For autologous proliferation assays, CD14-depleted PBMC fractions were co-cultured with mock- or PPRV-infected DC pulsed with the T cell superantigen SEB (1 µg/mL) seeded directly in the well (direct contact) or in a transwell insert (transwell) (Corning 3 µm pores). For IL-10 inhibition assays, cells were co-cultured in presence of 5 µg/mL anti-IL-10 blocking antibody (clone CC320; Novus Biologicals) or 5 µg/mL mouse IgG1 isotype control. After 96-h coculture, cells were transferred to V-bottom 96-well plate and washed twice with PBS by centrifugation at 400 ×g for 3 min at 4°C. Cell viability was assessed by staining with Live/Dead fixable near-infrared (IR) dead cell stain kit (Thermo Fisher) (1:1000 dilution in PBS, 50 µL/well) for 15 min on ice, followed by one wash with PBS stain buffer and subsequently labeled with anti-ovine CD4 (clone 44.38; Bio-Rad) and anti-ovine CD8 (clone 38.65; Bio-Rad) antibodies. Appropriate unlabeled, isotype, and fluorescence-minus-one controls were used for cell gating. Data acquisition was performed on a FACSCelestaSORPcytometer (BD Biosciences), and analysis was performed with FlowJo v.10 software by gating on live CD4^+^ or CD8^+^ positive events.

### Quantitative PCR

Transcription levels of *Tfnα, Ifnα*, *IL-10, IL-1β, IL-12, IL-6, CCL2* and *β-actin* genes were evaluated by RT-qPCR using SYBR Green I Master Reagents (Roche). Primer details can be found in [21]. All PCRs were performed on a Light Cycler 480 System instrument (Roche). Quantification of ovine transcripts was determined by normalizing gene expression to *β-actin* gene expression, and relative expression levels were calculated using the 2^−ΔΔCt^ method [22], where ∆∆Ct = Sample ∆Ct average – Control group (mock) ∆Ct average.

### Multiplex cytokine assay

Culture supernatants of mock- or PPRV-infected MoDC at 48 hours post-infection were collected from 5 goats and concentration of TNF-α, IL-1α, IL-6, IL-10, IL-4, and CCL4 measured using the MILLIPLEX® Ovine Cytokine/Chemokine Panel 1 (Millipore) according to the manufacturer’s protocol. Sample acquisition was performed on a MAGPIX instrument (Luminex).

### Statistical analysis

Statistical analysis was carried out using Prism 8.0 software (GraphPad Software Inc). Normality was assessed with the Shapiro – Wilk test, and appropriate statistical tests were applied as indicated in figure legends. Significance was set at *p* ≤ 0.05.

## Results

### Generation and characterization of caprine monocyte-derives dendritic cells

**Figure 1.**
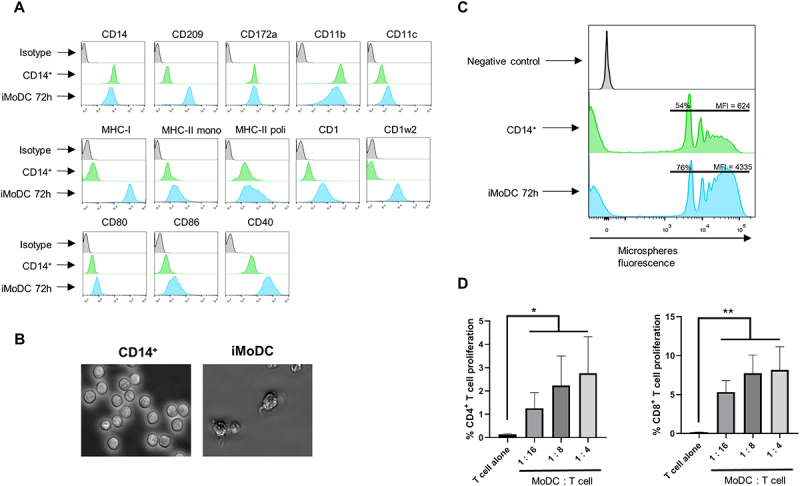
**Goat CD14^+^ monocyte differentiation to iMoDC. (A)** Representative histograms of cell surface markers measured by flow cytometry in monocytes (green) and differentiated iMoDC (blue) in 5 donor goats. Isotype controls were included (grey). **(B)** Microscopy images of freshly isolated monocytes (0 h) (left) and iMoDC cells (72 h) (right) (400X). **(C)** Microsphere phagocytosis assay assessed by flow cytometry, showing the percentage of cells and the geometric mean fluorescence intensity of beads (Geo MFI) captured by monocytes (green) compared to differentiated iMoDC (blue). As a control, the histogram for cultured iMoDC without microspheres is shown (grey). **(D)** Mixed lymphocyte reaction (MLR) assay of allogeneic MoDC and T cell used to assess T cell proliferation induced by MoDCs at different MoDC : T cell ratios by flow cytometry. A control in which only T cell (T cell alone) were cultured was used to evaluate the induced proliferation. (left panel) percentage of CD4^+^ T cell proliferation. (right panel) percentage of CD8^+^ T cell proliferation. * *p* < 0.05; ** *p* < 0.01; one way ANOVA with Fisher’s LSD post-test.

To generate functional goat monocyte-derived DCs (MoDCs), PBMCs were first isolated and CD14^+^ monocytes were subsequently enriched using magnetic cell sorting. The purified CD14^+^ monocytes (0 h) were then cultured in RPMI medium supplemented with IL-4 and GM-CSF to promote their differentiation into immature MoDCs (iMoDCs) over a period of 72 h. Phenotypic analyses by flow cytometry confirmed the differentiation process, as evidenced by the upregulation of surface markers characteristic of DCs [23] (Figure 1A). An increase in CD209 (DC-SIGN), molecules associated with PAMPs recognition, was observed when compared to monocytes. Similarly, the levels of co-stimulatory molecules CD80, CD86 and CD40 were also increased on differentiated cells. Classical MHC molecules, MHC-I and MHC-II, were upregulated, as were non-classical MHC molecules CD1 and CD1w2. Finally, the expression of integrin CD11c, whose expression is typical of DCs, increased in iMoDCs. In addition, these cells express the CD14, CD11b and CD172a markers typical of MoDCs. Morphological changes were assessed over the differentiation period. The most characteristic changes included the state of adherence, which changed from suspension for the monocytes (0 h) to semi-adherence for the iMoDC (72 h), the increase in size, and the change in shape showing less rounded cells and the presence of dendrites on the cell surface at 72 h (Figure 1B). The presence of all these morphological changes supports the flow cytometry data indicating the differentiation of monocytes to iMoDC with this protocol.

We next analyzed the phagocytic capability of iMoDCs, as immature DCs are known to exhibit greater phagocytic activity than monocytes [24]. To evaluate this, FluoSpheres microspheres (1 μm diameter, conjugated to the Crimson fluorochrome) were added to cell cultures, and bead uptake was measured by flow cytometry (Figure 1C). iMoDCs demonstrated significantly higher phagocytic activity compared to monocytes. These findings confirmed that the generated cells exhibited characteristic surface and functional features of iMoDCs, including enhanced phagocytic capability.

DCs act as sentinel immune cells, actively sampling their environment in the immature state. Upon encountering a pathogen, they undergo maturation, acquiring an enhanced ability to present antigens and activate T cells. To evaluate the antigen-presenting capacity of goat MoDC, we stimulated them with the TLR7/8 agonist R848 to induce maturation. Matured MoDCs (mMoDCS) were then co-cultured with CellTrace Violet-labeled allogeneic T cells for 96 h in a mixed lymphocyte reaction (MLR) assay. Flow cytometry analyses revealed that mMoDCs effectively induced the proliferation of both CD4^+^ and CD8^+^ T cells (Figure 1D), demonstrating their functional capacity as antigen-presenting cells (APC). Together, these results establish a robust protocol for the generation of functional goat MoDCs, which exhibit typical surface marker expression, strong phagocytic activity, and the ability to activate allogeneic T cells.

### PPRV infects CD14^+^ monocyte without impairing their differentiation into iMoDCs but reduces their phagocytic function

**Figure 2.**
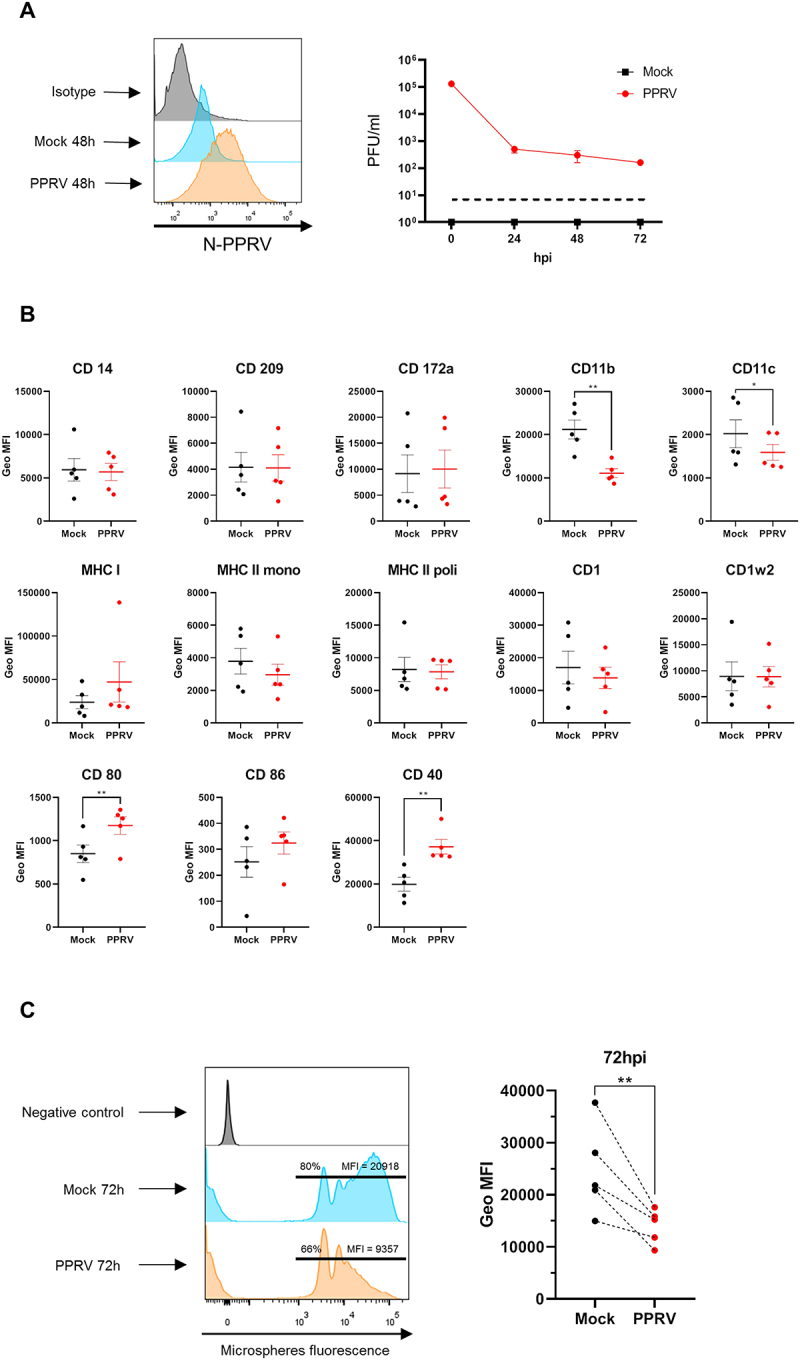
**CD14^+^ monocyte infection with PPRV does not prevent differentiation to iMoDC but impairs phagocytic activity**. Freshly isolated CD14^+^ cells were infected with PPRV at a MOI of 1 (or mock-infected as control) and differentiated into iMoDC for 72 h. **(A)** Representative flow cytometry intracellular staining histogram using anti-PPRV-N monoclonal antibody in mock-infected (blue) and PPRV infected (orange) cells. Isotype controls were included (gray) (left). Titration by plaque assays in VDS cell line of iMoDC culture supernatant at 0 (input), 24, 48, and 72 hpi (right). **(B)** Geometric mean fluorescence intensity (Geo MFI) of cell surface markers (mean ± SD) measured by flow cytometry in mock-infected (black) and PPRV-infected (red) iMoDC measured in 5 donor goats. *p < 0.05; **p < 0.01; multiple-ratio paired t test with Bonferroni-Dunn correction. **(C)** Microsphere phagocytosis assay assessed by flow cytometry. (left panel) Representative histograms showing a decrease in both the percentage of cells and the number of beads (Geo MFI) captured by PPRV-infected iMoDC (orange) compared to mock-infected iMoDC (blue). As a control, the histogram for iMoDC cultured without microspheres is shown (grey). (right panel) Geo MFI (mean ± SD) (*n* = 5) of PPRV-infected (red) and mock-infected (black) iMoDC in microsphere phagocytosis assay assessed by flow cytometry. **p < 0.01; ratio paired t test.

To investigate the impact of PPRV infection on monocyte function, we first assessed whether the virus was able to infect CD14^+^ monocytes. Cells were infected at the start of the culture (0 h) with the virulent PPRV ICV89´ strain, or mock-infected as a control, and then cultured for 72 hours to induce differentiation into iMoDC. At 48 hours post-infection (hpi), cells were harvested and stained with antibodies against the PPRV N protein. Flow cytometry analyses revealed a fluorescence shift in infected cultures compared to mock-infected controls, confirming intracellular viral presence at 48hpi (Figure 2A left). To determine whether the infection was productive, supernatants were collected at 0, 24, 48 and 72 hpi. Despite the detection of intracellular N protein, no infectious virus was detected in the supernatants, indicating a nonproductive infection (Figure 2A right).

We then evaluated the expression of surface markers on monocytes after 72 hours of differentiation. Flow cytometry showed that PPRV-infected cells exhibited increased expression of co-stimulatory molecules CD80 and CD40, while CD86 showed a similar trend without reaching statistical significance. In contrast, expression of integrins CD11b and CD11c was reduced in infected cells (Figure 2B). However, other characteristic iMoDCs markers remained unchanged between PPRV-infected and control cultures, suggesting that PPRV infection does not hinder monocyte differentiation into iMoDC. To assess functional consequences, we performed microsphere phagocytosis assays. Flow cytometry analyses revealed that PPRV-infected iMoDCs showed a reduced percentage of phagocytic cells and a lower bead uptake (geometric mean fluorescence intensity, Geo MFI) compared to mock-infected cells (Figure 2C). Taken together, these findings indicate that PPRV can infect CD14^+^ monocytes without disrupting their differentiation into iMoDCs, but it compromises the phagocytic function of the differentiated DC.

### PPRV infection of goat iMoDC is nonproductive and induces limited apoptosis

**Figure 3.**
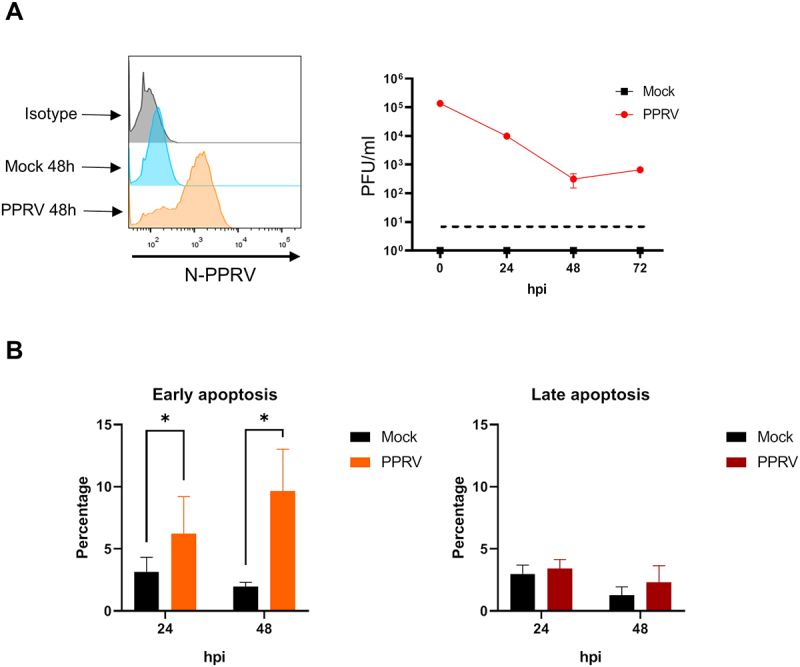
**PPRV infects goat iMoDC and does not produce major cell death**. iMoDC were infected with PPRV at an MOI of 1 (or mock-infected as control) and cultured for up to 72 h. **(A)** (left panel) Representative flow cytometry intracellular staining histogram using anti-PPRV-N monoclonal antibody in mock-infected (blue) and PPRV infected (orange) iMoDC. Isotype controls were included (gray). (right panel) titration by plaque assays in VDS cell line of iMoDC culture supernatant at 0 (input), 24, 48, and 72 hpi. **(B)** Percentage of iMoDC in early apoptosis state (left) and late apoptosis state (right) in mock (black) or PPRV (orange and brown)-infected MoDC cultures, at 24 and 48 hpi (*n* = 4). *, *p* < 0.05; paired t test.

To further explore the effects of PPRV on goat antigen-presenting cells, iMoDCs were infected at 48 hours of culture with the virulent PPRV ICV89´ strain. At 96 hours of culture (48hpi), cells were harvested and stained with antibodies against the PPRV N protein. Flow cytometry confirmed the presence of viral nucleoprotein in infected iMoDCs, indicating successful infection (Figure 3A left). To determine whether PPRV infection was productive, supernatants were collected at 0, 24, 48 and 72 hpi. As observed in monocyte cultures, no increase in viral titers was detected over time, indicating that PPRV infection in goat iMoDCs is nonproductive (Figure 3A right).

We next assess whether PPRV infection induces apoptosis in iMoDC. Annexin-V staining followed by flow cytometry at 24 and 48 hpi revealed a significant increase in early apoptotic cells in infected cultures compared to mock-infected controls. However, levels of late apoptosis remained unchanged (Figure 3B). These results suggest that PPRV triggers a modest apoptotic response in iMoDC, which is consistent with cell count data showing no significant differences between PPRV-infected and control iMoDC (data not shown).

### PPRV infection enhances co-stimulatory molecule expression and reduces phagocytic activity in goat iMoDCs

**Figure 4.**
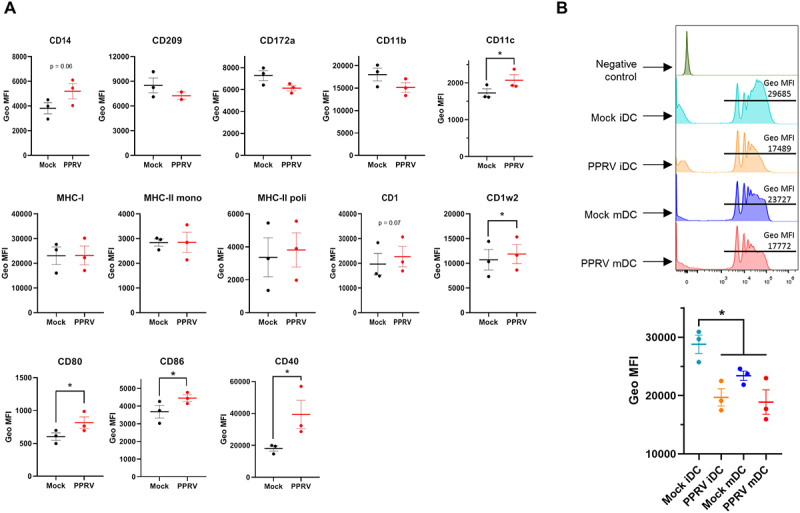
**PPRV infection in iMoDC increases activation marker expression and reduces phagocytosis. (A)** Geometric mean fluorescence intensity (Geo MFI) of cell surface markers (mean ± SD) measured by flow cytometry in mock-infected (black) and PPRV-infected (red) iMoDC measured in 3 donor goats. *p < 0.05; multiple-ratio paired t test with Bonferroni-Dunn correction. **(B)** (top panel) microsphere phagocytosis assay assessed by flow cytometry, showing a decrease in both the percentage of cells and the number of beads (Geo MFI) captured by PPRV-infected iMoDC (orange) and mMoDC (purple) compared to mock-infected iMoDC (blue) and mMoDC (red). As a control, the histogram for iMoDC cultured without microspheres is shown (green). (bottom panel) Geo MFI (mean ± SD) (*n* = 3) of PPRV-infected (orange and red) and mock-infected (blue and purple) MoDC in microsphere phagocytosis assay assessed by flow cytometry are plotted. *p < 0.05, One-way ANOVA with Fisher’s LSD post-test.

To further characterize the impact of PPRV on goat iMoDCs, we analyzed the expression of surface markers by flow cytometry at 48hpi. Infected iMoDCs exhibited a significant increase in the geometric mean fluorescence intensity of the co-stimulatory molecules CD80, CD86 and CD40 compared to mock-infected controls. Additionally, expression of the integrin CD11c was significantly elevated in infected cells. Notably, we also observed increased expression of the non-classical MHC molecules CD1 and CD1w2 (Figure 4A). These findings suggest that PPRV infection may promote partial activation or maturation of iMoDCs. To assess whether this phenotypic activation was accompanied by functional changes, we performed microsphere-based phagocytosis assays. Infected iMoDCs showed a marked reduction in both the percentage of cells and the number of beads internalized per cell, as measured by geometric mean fluorescence intensity, compared to mock-infected iMoDC (Figure 4B). When mock-infected MoDC were matured with R848, we observed a similar decrease in phagocytic activity, which confirmed that PPRV infection drives MoDC activation. These results are consistent with those observed in PPRV-infected monocytes and support the notion that PPRV infection induces an activated DC phenotype.

### PPRV infection impairs the antigen-presenting capacity of MoDcs

**Figure 5.**
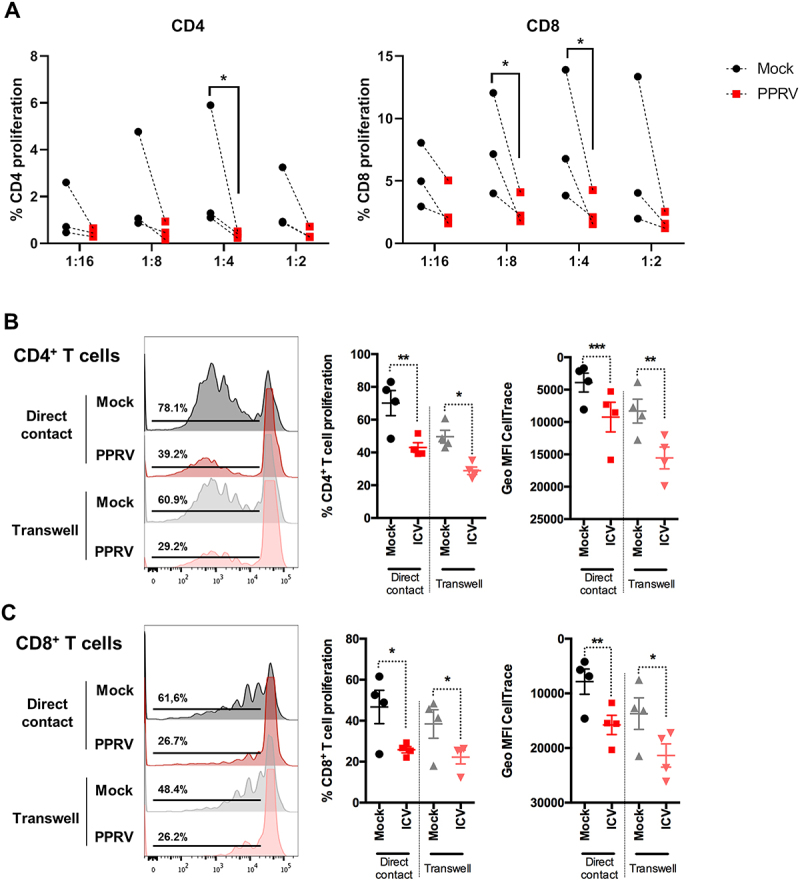
**PPRV-infected DC reduces T cell proliferation through direct contact and soluble factors. (A)** Allogeneic MLR with T cells and MoDC. T cells were labelled with CellTrace violet to evaluate proliferation and cultured with mock- or PPRV-infected allogeneic MoDC at different ratio for 120 h. Cells were then labelled with anti-CD4 and anti-CD8 antibodies and proliferation evaluated in each T cell population by flow cytometry. Percentage of CD4^+^ T cell (left panel) and CD8^+^ T cell (right panel) proliferation in 3 independent experiments in 3 different goat donor. *p < 0.05 multiple ratio paired t-test with Bonferroni-Dunn correction. **(B and C)** autologous CD14-depleted PBMC labeled with CellTrace violet were co-cultured in presence of SEB with mock- or PPRV-infected DC seeded directly in the well (direct contact) or in a transwell insert (transwell). After 4 days, cells were stained for CD4 and CD8 and proliferation analyzed by flow cytometry. Representative histograms of **(B)** CD4^+^ and **(C)** CD8^+^ T cell proliferation in direct contact or transwell co-cultures. Percentage and geometric mean CellTrace violet fluorecence intensity in **(B)** CD4^+^ and **(C)** CD8^+^ T cells direct contact and transwell co-cultures with mock- or infected-PPRV in 4 donor goat. *p < 0.05; **p < 0.01; ***p < 0.001, One-way ANOVA with Fisher’s LSD post-test.

To evaluate the effect of PPRV infection on the antigen-presenting function of MoDCs, we conducted allogeneic mixed lymphocyte reaction (MLR) assays. Matured MoDCs, either mock- or PPRV-infected, were co-cultured with allogeneic T cells. Flow cytometry analysis revealed a significant reduction in the proliferation of both CD4^+^ and CD8^+^ T cells in cultures with PPRV-infected MoDCs (Figure 5A), indicating impaired antigen presentation. This impairment was further confirmed using an autologous co-culture system stimulated with the T cell superantigen SEB. In this setting, PPRV-infected matured MoDC induced lower levels of IFN-γ production in CD4^+^ T cells compared to mock-infected controls (Supplementary Figure S2). These data therefore indicate that PPRV infection is only driving a partial maturation of MoDC, since their antigen-presenting capacity is impaired.

To investigate the mechanisms underlying this suppression, we performed autologous SEB-stimulated proliferation assays using two culture conditions: direct contact and transwell separation. In both systems, PPRV-infected matured MoDCs significantly reduced CD4^+^ and CD8^+^ T cell proliferation (Figure 5B and C), suggesting that the impairment of T cell activation is mediated by a combination of direct cell-to-cell interactions and soluble inhibitory factors.

### PPRV infection impairs DC-T cell conjugation and promotes IL-10 production to suppress T cell activation

**Figure 6.**
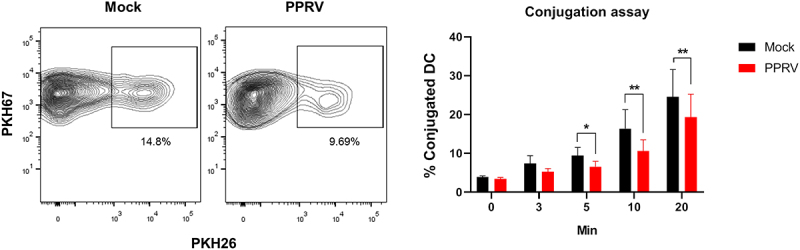
**PPRV infection impairs MoDC conjugation to immune cells**. Conjugation assays of MoDC and autologous CD14^−^ PBMC cells. MoDC were mock- or PPRV-infected for 48 hours. MoDC were labeled with PKH67 and pulsed with the superantigen SEB to facilitate conjugation. The autologous CD14^−^ PBMC fraction was labeled with PKH26. MoDC and CD14^−^ PBMC were co-cultured and fixed with 4% PFA at the indicated times prior to analysis by flow cytometry of PKH67^+^ PKH26^+^ doublets indicative of cell conjugation. Representative contour plots of PKH67-labeled DC conjugated to PKH26-labeled PBMC after 10 min incubation are shown. Percentage of MoDC conjugated to CD14^−^ PBMC are plotted (mean ± SEM) (*n* = 3). *p < 0.05, **p < 0.01 multiple ratio paired t-test with Bonferroni-Dunn correction.

To further investigate the mechanisms by which PPRV impairs T cell activation, we first assessed the ability of mock- or PPRV-infected MoDC to form conjugates with autologous immune cells, a critical step for the establishment of immunological synapses. Matured MoDCs were labeled with the fluorescent cell linker PKH67, and autologous CD14^−^ PBMC were labeled with the fluorescent cell linker PKH26. The formation of doublets was analyzed by flow cytometry at 0, 3, 5, 10, and 20 min of co-incubation (Figure 6). PPRV-infected MoDCs showed a reduced capacity to form conjugates with target cells compared to mock-infected controls, suggesting that viral infection may disrupts the physical interactions necessary for effective antigen presentation.

**Figure 7.**
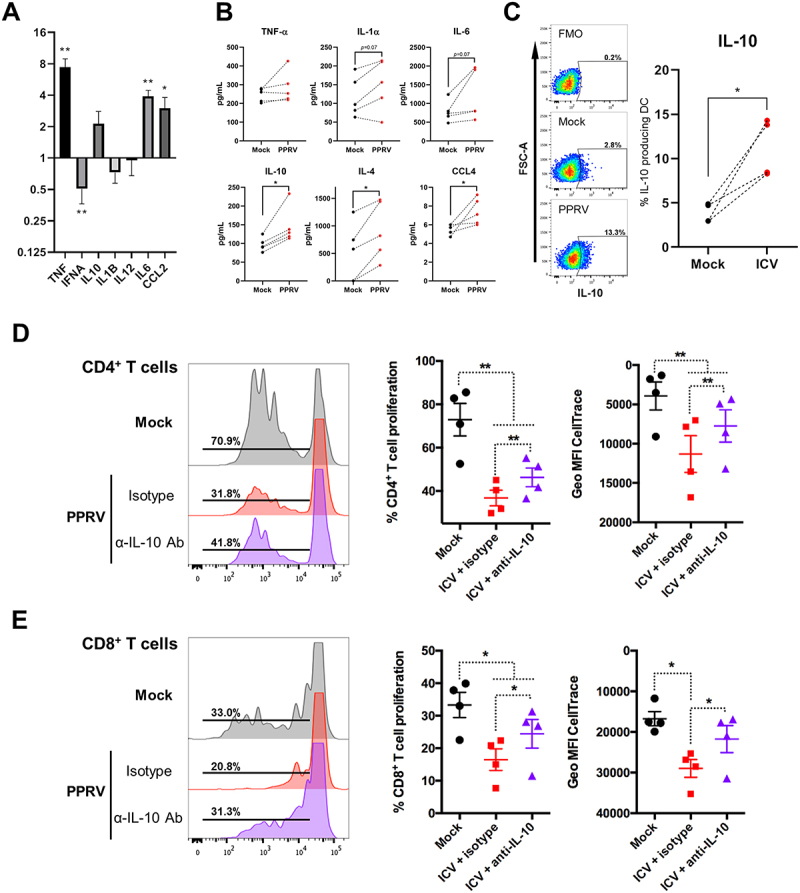
**IL-10 contributes to the inhibition of T cell activation by PPRV-infected DCs. (A)** Quantification of *TNF*, *IFNA*, *IL10*, *IL1B*, *IL12*, *IL6* and *CCL2* transcripts by qPCR. Cell cultures were harvested for RNA isolation and RT-qPCR quantification of *TNF*, *IFNA*, *IL10*, *IL1B*, *IL12*, *IL6* and *CCL2* transcripts. Data of four goats (*n* = 4) are represented as relative expression using the 2^−∆∆Ct^ method normalized to mock. *p < 0.05; **p < 0.01. Statistical analysis was carried out with two-tailed unpaired t test. **(B)** Cell culture supernatant from PPRV- or mock-infected DC were collected at 48hpi and cytokine concentration (TNF-α, IL-1α, IL-6, IL-10, IL-4, and CCL4) measured by Milliplex assay. Cytokine concentration in 5 different independent goat DC cultures are plotted. * *p* < 0.05, paired Student’s t test. **(C)** Representative dot-plots for IL-10 in CD40-gated mock- or PPRV-infected DC. Fluorescence minus one (FMO) channel was used for IL-10 gating. Percentage of IL-10^+^ DC in mock and PPRV-infected DC are plotted. * *p* < 0.05, paired Student’s t test. **(D and E)** autologous CD14-depleted PBMC labeled with CellTrace violet were stimulated with SEB and co-cultured with mock- or PPRV-infected DC in presence of anti-IL-10 blocking antibody or an isotype control. After 4 days, cells were stained for CD4 and CD8 and proliferation analyzed by flow cytometry. Representative histograms of **(D)** CD4^+^ and **(E)** CD8^+^ T cell proliferation in mock or PPRV-infected DC co-cultures in presence of blocking IL-10 antibody or isotype. Percentage and geometric mean CellTrace violet fluorescence intensity in **(D)** CD4^+^ and **(E)** CD8^+^ T cells in co-cultures with mock- or infected-PPRV treated with isotype or IL-10 blocking antibody in 4 donor goats. *p < 0.05; **p < 0.01, One-way ANOVA with Fisher’s LSD post-test.

To identify soluble mediators involved in the suppression of T cell responses, we quantified the expression of several cytokine genes (TNF, IFNA, IL10, IL1B, IL12, IL6 and CCL2) in infected iMoDCs. Gene expression levels were normalized to mock-infected controls. PPRV infection led to increased expression of TNF, IL10, IL6 and CCL2, while IFNA expression was reduced (Figure 7A). Although IL10 mRNA levels showed an upward trend, the increase did not reach statistical significance. To further evaluate changes in cytokine production we measured by multiplex assays the concentration of TNF-α, IL-1α, IL-6, IL-10, IL-4, and CCL4 present in iMoDC 48 hours after PPRV infection (Figure 7B). We found a significant increase in IL-4, CCL4 and IL-10 production after PPRV infection, while secretion of pro-inflammatory cytokines TNF-α, IL-1α, and IL-6 tended to increase when compared to mock-infected cells. These data indicate that infection is leading to the production of pro-inflammatory cytokines, but that it also triggers immunomodulatory cytokine secretion in MoDCs.

Given IL-10 immunomodulatory role, we next evaluated IL-10 protein production by flow cytometry to confirm the multiplex finding (Figure 7C). PPRV-infected iMoDCs exhibited elevated levels of IL-10, a cytokine known to modulate T cell responses and promote immune tolerance [25]. To assess the functional role of IL-10 in the observed T cell suppression, we performed co-culture assays using SEB-stimulated autologous T cells in the presence or absence of an IL-10 blocking antibody (Figure 7D and E). Blocking IL-10 partially restored CD4^+^ and CD8^+^ T cell proliferation in cultures with PPRV-infected matured MoDCs, implicating IL-10 as a key mediator of the immunosuppressive effects induced by the virus. Taken together, these findings indicate that PPRV impairs T cell activation through a dual mechanism: by disrupting DC-T cell conjugate formation and by inducing the production of immunomodulatory cytokines such as IL-10.

## Discussion

The present study provides the first comprehensive characterization of PPRV infection in goat MoDCs, revealing how the virus modulates their phenotype and impairs their immunological function. A central and novel finding of this work is the upregulation of the immunosuppressive cytokine IL-10 in PPRV-infected MoDCs, which appears to play a pivotal role in dampening the host immune response.

Given the low frequency of DCs in peripheral blood, we employed an optimized protocol to generate MoDCs from CD14^+^ monocytes. This model is highly relevant, as monocyte to DC differentiation is a physiological process that occurs during infection and inflammation [26], making it a suitable system to study host-pathogen interactions. Our results demonstrate that, after isolation of monocytes and their subsequent differentiation and maturation, fully functional goat MoDCs were obtained. Goat MoDC had characteristic morphology, expressed typical membrane markers (CD209, CD172a, CD11b, CD11c, MHC-I, MHC- II, CD1, CD1w2, CD80, CD86 and CD40) and showed high phagocytic capacity. These results agree with those described in other MoDC differentiation assays in other ruminants [16,27,28].

Monocytes are known to be susceptible to morbillivirus infection [16,29]. Nevertheless, dendritic cells may play a pivotal role in *in vivo* infection by PPRV, as they can influence in viral particles distribution to lymph nodes [30]. Blood transcriptome analysis has recently shown that PBMC can be enriched in activated dendritic cells during PPRV infections in goats [31]. Dendritic cells are also important target cells for other morbilliviruses, as they have been described to be affected by infection and reduce their antigen presentation capacity [32]. In this regard, our findings demonstrate that PPRV can infect both goat monocytes and goat MoDCs without impairing their differentiation. Although PPRV infection induces increased expression of co-stimulatory molecules (CD80, CD86 and CD40) and non-classical MHC molecules (CD1, CD1w2), suggesting a partially activated or matured phenotype, this apparent activation does not translate into enhanced functionality. PPRV-infected MoDCs showed significantly reduced phagocytic activity consistent with a matured phenotype, but displayed a marked impairment in their ability to stimulate T cell proliferation, both in allogeneic and autologous settings. These observations are consistent with previous studies in sheep [16]. Importantly, while PPRV can infect caprine MoDCs, the infection does not result in productive viral replication, a phenomenon also reported for other morbilliviruses such as measles virus in human MoDC [33]. The upregulation of maturation markers may reflect a shift from antigen uptake to antigen presentation, a hallmark of DC maturation. However, this shift appears to be exploited by the virus. Similar to measles virus, which uses infected DCs to enhance lymphocyte infection [34], PPRV may leverage the antigen-presenting capabilities of partially matured DCs to facilitate viral dissemination, a “Trojan horse” strategy observed in other members of the *Paramyxoviridae* family [34]. Supporting this hypothesis, *in vitro* studies using goat PBMCs have shown that PPRV infection leads to transcriptional activation of DCs [35], reinforcing the notion that the virus modulates DC function to its advantage. Nevertheless, *in vivo* data suggest that monocyte infection is likely transient, as these cells are no longer infected with PPRV at day 9 post infection [36], indicating that PPRV may use monocytes as vehicles for early viral spread through the lymph nodes and subsequent lymphocyte infection.

Allogeneic MLRs revealed that PPRV infection significantly impairs the ability of MoDCs to stimulate both CD4^+^ and CD8^+^ T cell proliferation. This profound suppression mirrors the effects observed in measles virus infections, where DCs lose their capacity to activate naïve CD4^+^ T cells [37]. Similarly, previous studies have shown that PPRV-infected ovine MoDCs exhibit reduced T cell stimulatory function [16]. Our results obtained from the transwell assay indicate that this inhibition is mediated by both cell-to-cell contact and soluble factors. Crucially, we observed a marked decrease in the formation of stable conjugates between infected DCs and T cells, suggesting a disruption in the establishment of the immunological synapse, a critical interface for effective antigen presentation and T cell activation [38]. This disruption likely represents a key immune evasion strategy employed by PPRV. Indeed, other morbilliviruses such as measles virus are known to actively inhibit immunological synapse formation between DCs and T cells [39]. The inability of infected DCs to form proper synapses may underlie the observed defects in T cell proliferation, highlighting a pivotal mechanism by which PPRV subverts host immunity. By targeting this fundamental step in immune communication, PPRV not only dampens the adaptive response but may also facilitate systemic viral dissemination.

*In vitro* infection of goat MoDCs by PPRV leads to the upregulation of several proinflammatory cytokine genes, including *TNF*, *IL6* and *CCL2*, which are typically associated with viral infections [16] and DC maturation [40]. Milliplex assays confirmed that pro-inflammatory cytokines tended to be elevated in the culture supernatant of infected DC. However, this inflammatory response is paradoxically accompanied by a significant downregulation of *IFNA*, a key antiviral cytokine. This suppression aligns with previous findings showing that PPRV effectively inhibits type I interferon responses both *in vitro* and *in vivo* [16,41,42], potentially compromising the host’s ability to mount an effective antiviral response despite the apparent activation of innate immune sensors. Curiously, we detected a significant increase in IL-4 in the culture supernatant of PPRV-infected DC when compared to mock. IL-4 is mostly produced by T cells, but its production can be induced on dendritic cells exposed to IL-4 [43], as in our case. IL-4 promotes Th2 differentiation, which is not optimal for antiviral responses and it would be interesting in future work to evaluate whether PPRV-infection is skewing the Th1/Th2 balance to evade immunity.

Strikingly, we also observed a robust increase in the expression of the immunomodulatory cytokine IL-10 [9], both at gene and protein levels, in PPRV-infected goat MoDCs. This cytokine plays a central role in immune regulation, particularly in dampening inflammatory responses and inhibiting antigen-presenting cell function. Similar IL-10 upregulation has been reported in canine MoDC infected with canine distemper virus [44], another morbillivirus, suggesting a conserved immunoevasive strategy within the viral family. The immunosuppressive effects of IL-10 are well documented, including its ability to inhibit macrophage-dependent T cell proliferation [45]. In our study, *in vitro* blockade of IL-10 partially restored T cell activation, directly implicating IL-10 in the suppression of adaptive immune responses mediated by PPRV-infected goat MoDCs. A similar mechanism has been described in other viral infections, such as foot-and-mouth disease virus in swine [46], where IL-10 contributes to the establishment of a tolerogenic environment. Thus, these results underscore the critical role of IL-10 as a central mediator of immune suppression during PPRV infection. By inducing IL-10 expression, PPRV not only disrupts the antiviral cytokine milieu but also actively reprograms DCs toward a tolerogenic phenotype, impairing their ability to activate T cells. The mechanisms through which the virus reprograms DCs to produce IL-10 remain to be determined, but it could implicate the modulation of signaling pathways such PI3K/Akt by the infection [47,48], which could result in limited DC activation that in turn could promote the tolerogenic phenotype. This immunomodulatory strategy, combined with the inhibition of conjugate formation and reduced IFN signaling, reveals a multilayered viral tactic to evade host immunity and promote viral dissemination.

In conclusion, PPRV infection affects goat DCs activity. Partial maturation of these cells occurs during infection, as evidenced by a reduction in phagocytic capacity and an increase in activation membrane markers, such as CD80 or CD40, but with an impaired antigen presentation capacity. However, the infection impairs DC antigen-presenting capacity through both cell-to-cell contact and soluble factors, including IL-10. Taken together, these mechanisms likely contribute to the immunosuppressive state induced by the disease in the host and may explain the delayed T cell responses typically observed in experimental infections in the natural host [49,50]. A deeper understanding of PPRV-induced immunosuppression in highly susceptible hosts like goats could support the development of more effective strategies to control this emerging transboundary threat.

## Ethical statement

All the animal experiments were carried in strict accordance with the recommendations in the guidelines of the Code for Methods and Welfare Considerations in Behavioral Research with Animals (Directive 86/609EC; RD1201/2005), and all efforts were made to minimize suffering. Experiments were approved by the Committee on the Ethics of CSIC and the National Animal Welfare Committee (PROEX 295.6/21).

## Supplementary Material

Supplemental Material

## Data Availability

The data that support the findings of this study are available in Digital.CSIC at http://hdl.handle.net/10261/399488 [51].
