# Supplementary figures and images for: Peste des petits ruminants virus (PPRV) modulates caprine dendritic cell function and induces immunosuppression through IL-10 upregulation
Source: Virulence. 2026 Feb 16;17(1):2629119. doi: 10.1080/21505594.2026.2629119 (PMC12915780; doi:10.1080/21505594.2026.2629119)

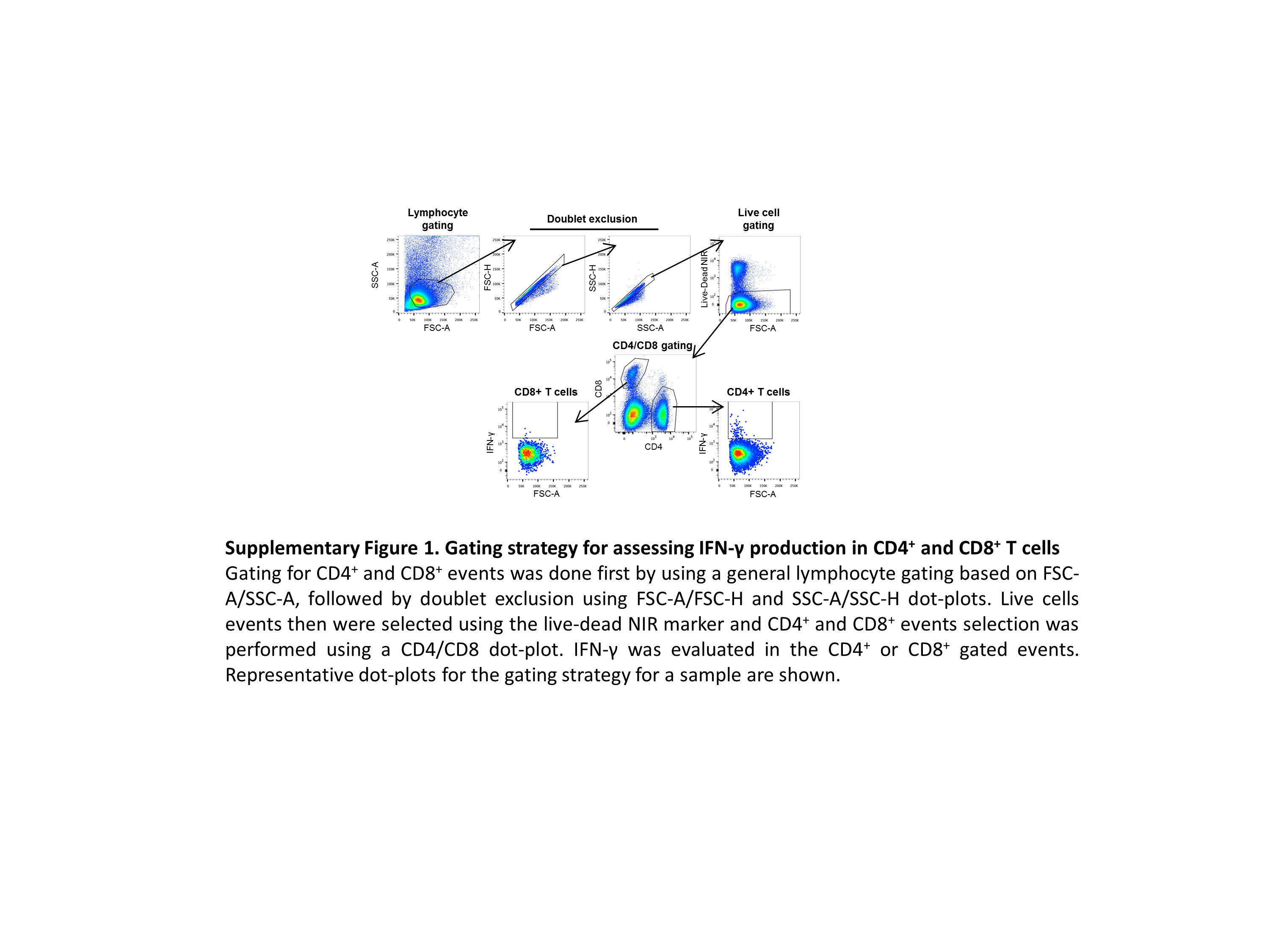

Supplement: Supplemental Material [file KVIR_A_2629119_SM8158.zip › New Suppl Fig 1.tif]

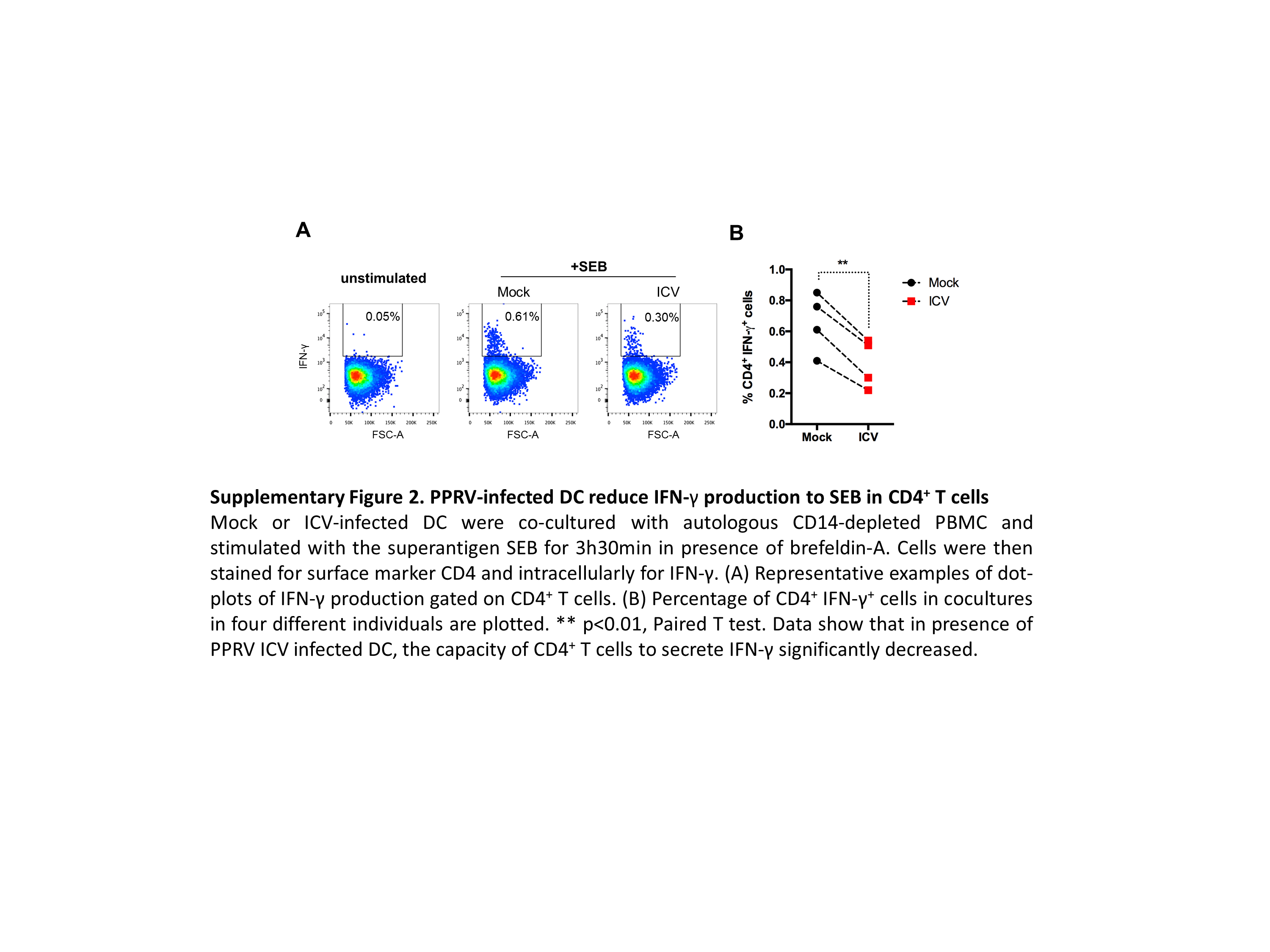

Supplement: Supplemental Material [file KVIR_A_2629119_SM8158.zip › New Suppl Fig 2.tif]
